# Supplementary material for: Insulin–Mimetic Dihydroxanthyletin-Type Coumarins from Angelica decursiva with Protein Tyrosine Phosphatase 1B and α-Glucosidase Inhibitory Activities and Docking Studies of Their Molecular Mechanisms
Source: Antioxidants (Basel). 2021 Feb 15;10(2):292. doi: 10.3390/antiox10020292 (PMC7919472; doi:10.3390/antiox10020292)
Supplement: Supplementary file 1 [file antioxidants-10-00292-s001.pdf]

Supplementary materials

Insulin–mimetic dihydroxanthyletin-type coumarins from *Angelica decursiva* with protein tyrosine phosphatase 1B and α-glucosidase inhibitory activities and docking studies of their molecular mechanisms

Md Yousof Ali [1], Susoma Jannat [2], Hyun Ah Jung [3] and Jae Sue Choi [4*]

[1] Department of Physiology and Pharmacology, Hotchkiss Brain Institute and Alberta Children's Hospital Research Institute, Cumming School of Medicine, University of Calgary, Calgary, AB T2N 4N1, Canada; mdyousof.ali@ucalgary.ca

[2] Department of Biochemistry and Molecular Biology, University of Calgary, T2N 4N1 Alberta, Canada; jannatacct@gmail.com

[3] Department of Food Science and Human Nutrition, Jeonbuk National University, Jeonju 54896, Korea

[4] Department of Food and Life Science, Pukyong National University, Busan 48513, Korea;

* Correspondence: jungha@jbnu.ac.kr (H.A.J); choijs@pknu.ac.kr; Tel.: +82-51-629-7547 (J.S.C.)

Supplymentary materils

Table of Contents

Figure S1. Dixon and Lineweaver-Burk plots for PTP1B inhibition of dihydroxanthyletin-type coumarins

Figure S2. Dixon and Lineweaver-Burk plots for α-glucosidase inhibition of dihydroxanthyletin-type coumarins

Figure S3. Molecular docking models for PTP1B inhibition of dihydroxanthyletin-type coumarins

Figure S4. Molecular docking models for α-glucosidase inhibition of dihydroxanthyletin-type coumarins

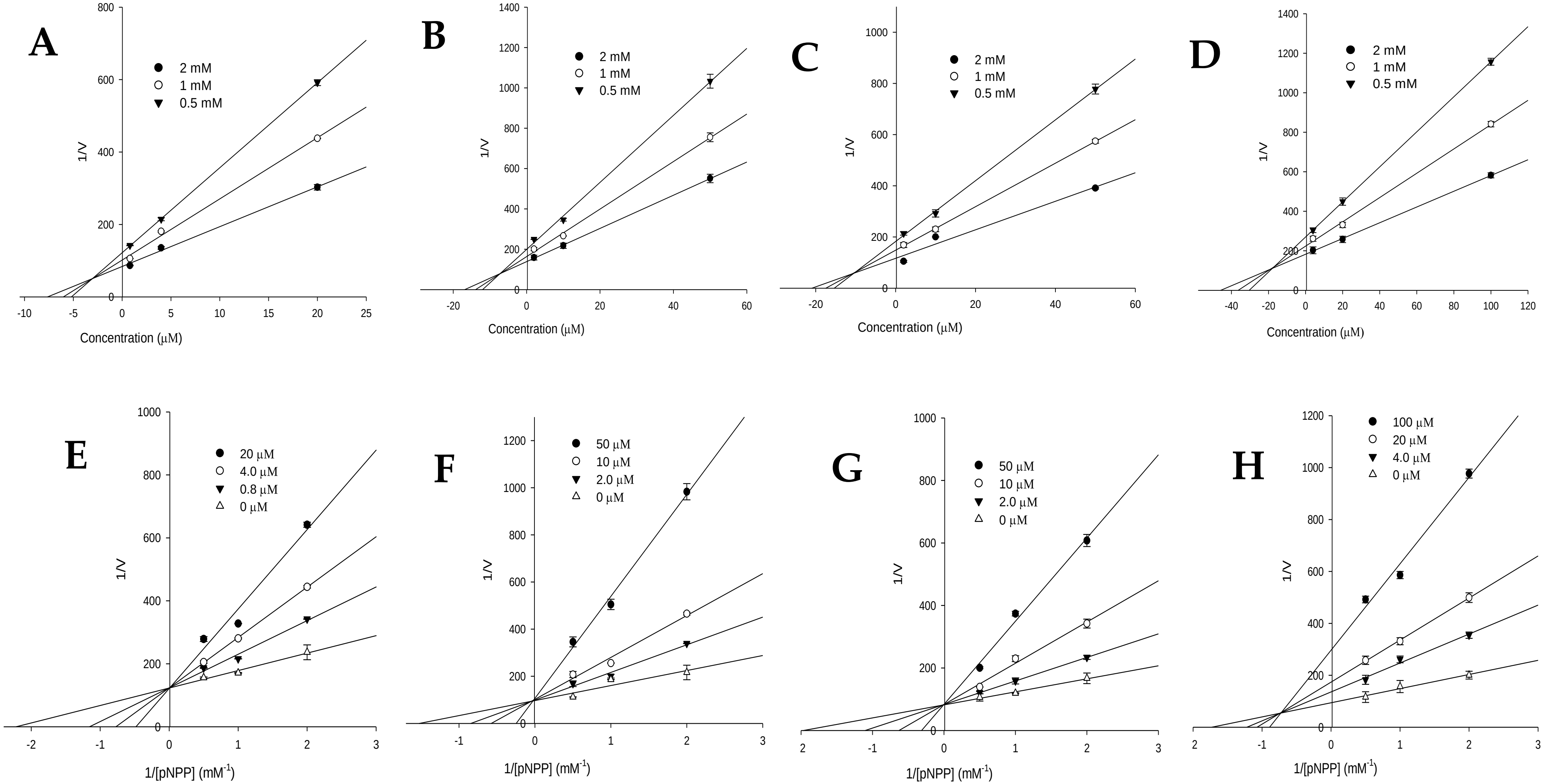

Figure S1. Dixon plots for PTP1B inhibition of dihydroxanthyletin-type coumarins. (+)-*trans*-decursidinol (A), Pd-C-I (B), Pd-C-II (C), Pd-C-III (D), were tested in the presence of different concentration of substrate (pNPP): 2mM (●); 1mM (○) and 0.5 mM (▼). Lineweaver-Burk plot for PTP1B inhibition of coumarins. PTP1B inhibition was analyzed in the presence of different concentration of sample as follows: 0 μM (Δ), 0.8 μM (▼), 4.0 μM (○) and 20 μM (●) for (+)-*trans*-decursidinol (E); 0 μM (Δ), 2 μM (▼), 10 μM (○) and 50 μM (●) for Pd-C-I (F); 0 μM (Δ), 2 μM (▼), 10 μM (○) and 50 μM (●) for Pd-C-II (G); 0 μM (Δ), 4 μM (▼), 20 μM (○) and 100 μM (●) for Pd-C-III (H).

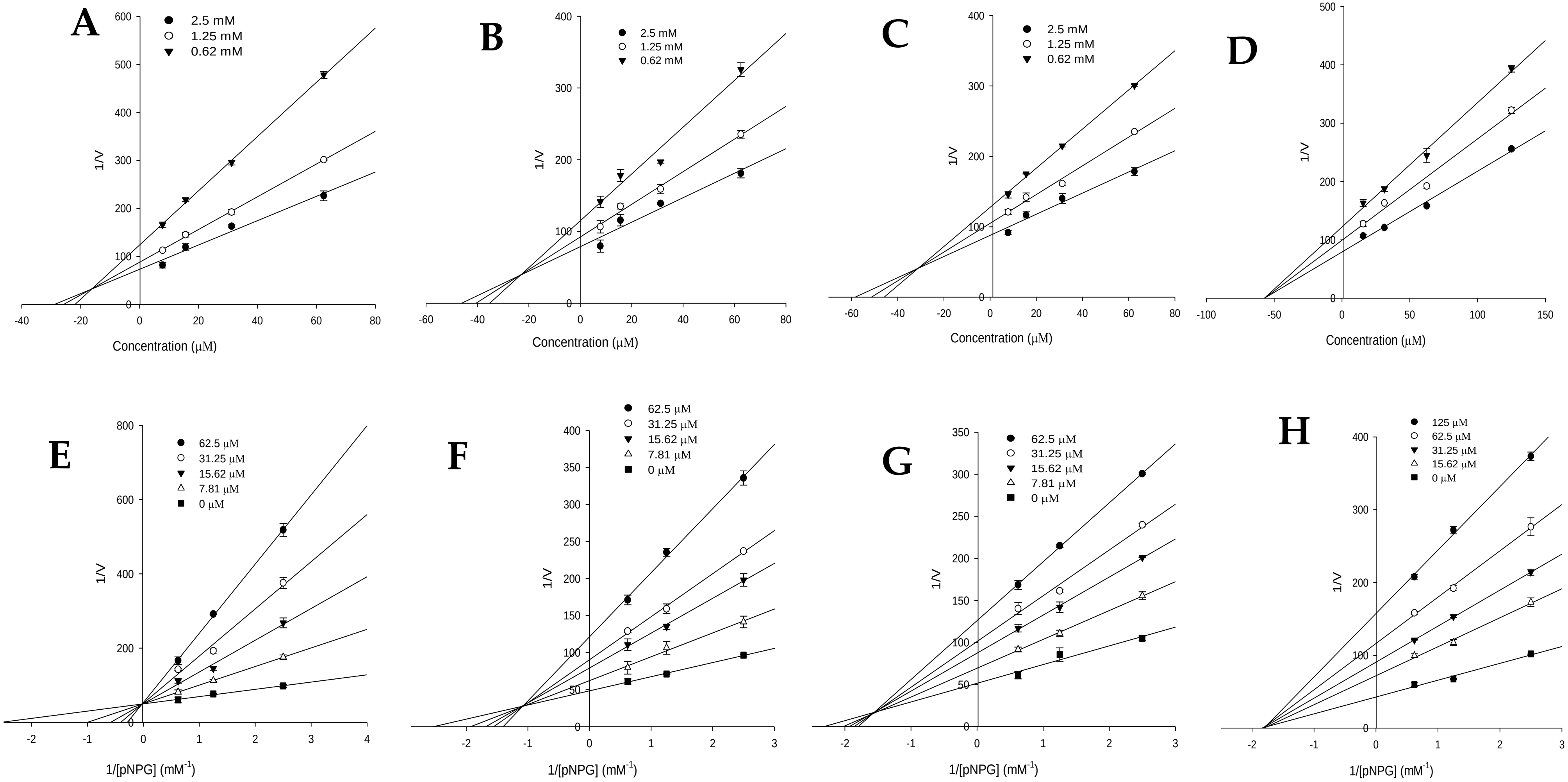

Figure S2. Dixon plots for α-glucosidase inhibition of dihydroxanthyletin-type coumarins. (+)-*trans*-decursidinol (A), Pd-C-I (B), Pd-C-II (C), Pd-C-III (D) were tested in the presence of different concentration of substrate (pNPG): 2.5mM (●); 1.25mM (○) and 0.625 mM (▼). Lineweaver-Burk plot for α-glucosidase inhibition of coumarins. α-Glucosidase inhibition was analyzed in the presence of different concentration of sample as follows: 0 μM (■), 7.81 μM (Δ), 15.62 μM (▼), 31.25 μM (○) and 62.5 μM (●) for (+)-*trans*-decursidinol (E); 0 μM (■), 7.81 μM (Δ), 15.62 μM (▼), 31.25 μM (○) and 62.5 μM (●) for Pd-C-I (F); 0 μM (■), 7.81 μM (Δ), 15.62 μM (▼), 31.25 μM (○) and 62.5 μM (●) for Pd-C-II (G); 0 μM (■), 15.62 μM (Δ), 31.25 μM (▼), 62.5 μM (○) and 125 μM (●) for Pd-C-III (H).

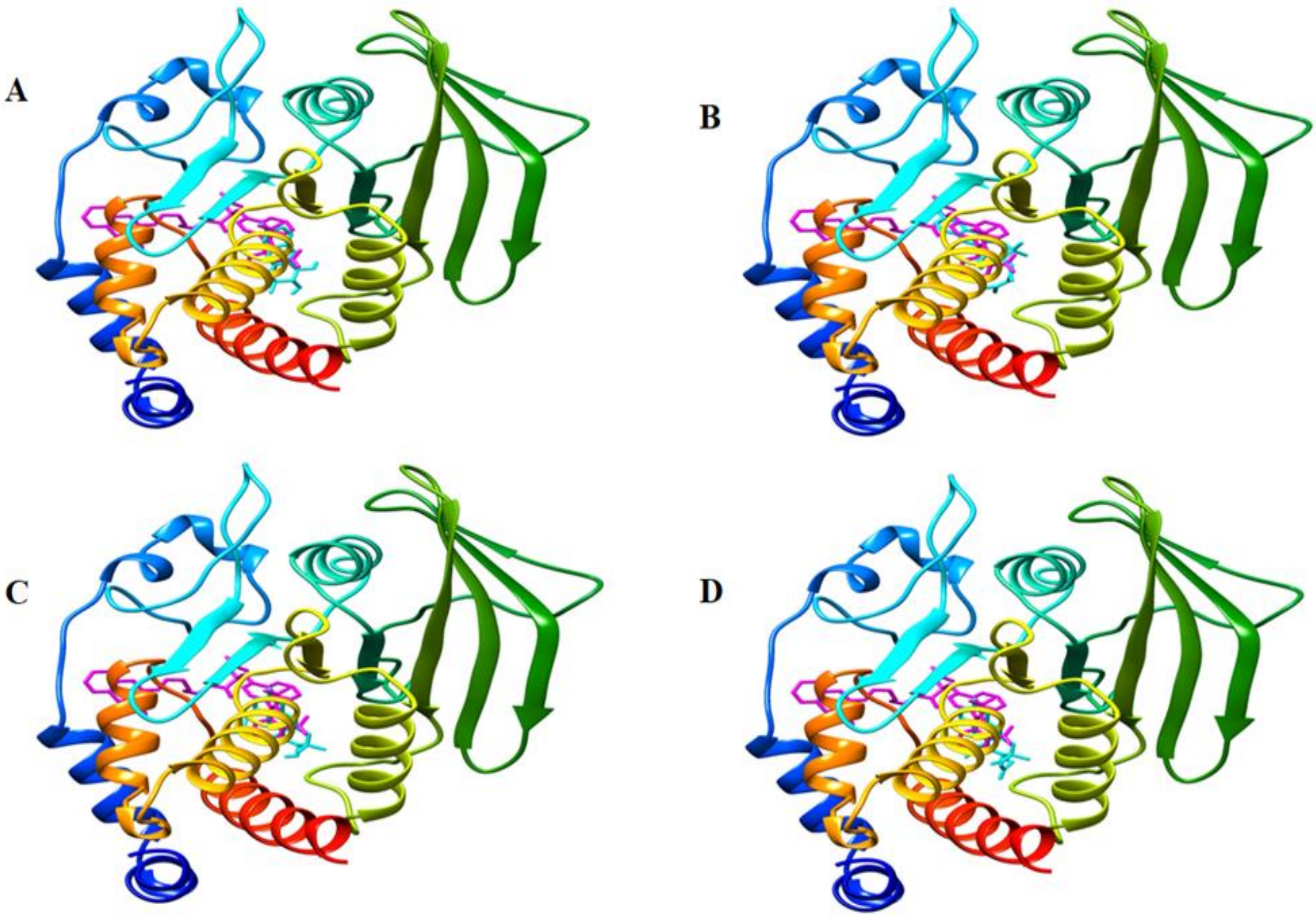

Figure S3. Molecular docking models for PTP1B inhibition of (+)-trans-decursidinol (A), Pd-C-I (B), Pd-C-II (C), and Pd-C-III (D). Compound 23 denote (magenta color) and dihydroxanthyletin-type coumarins (light blue color).

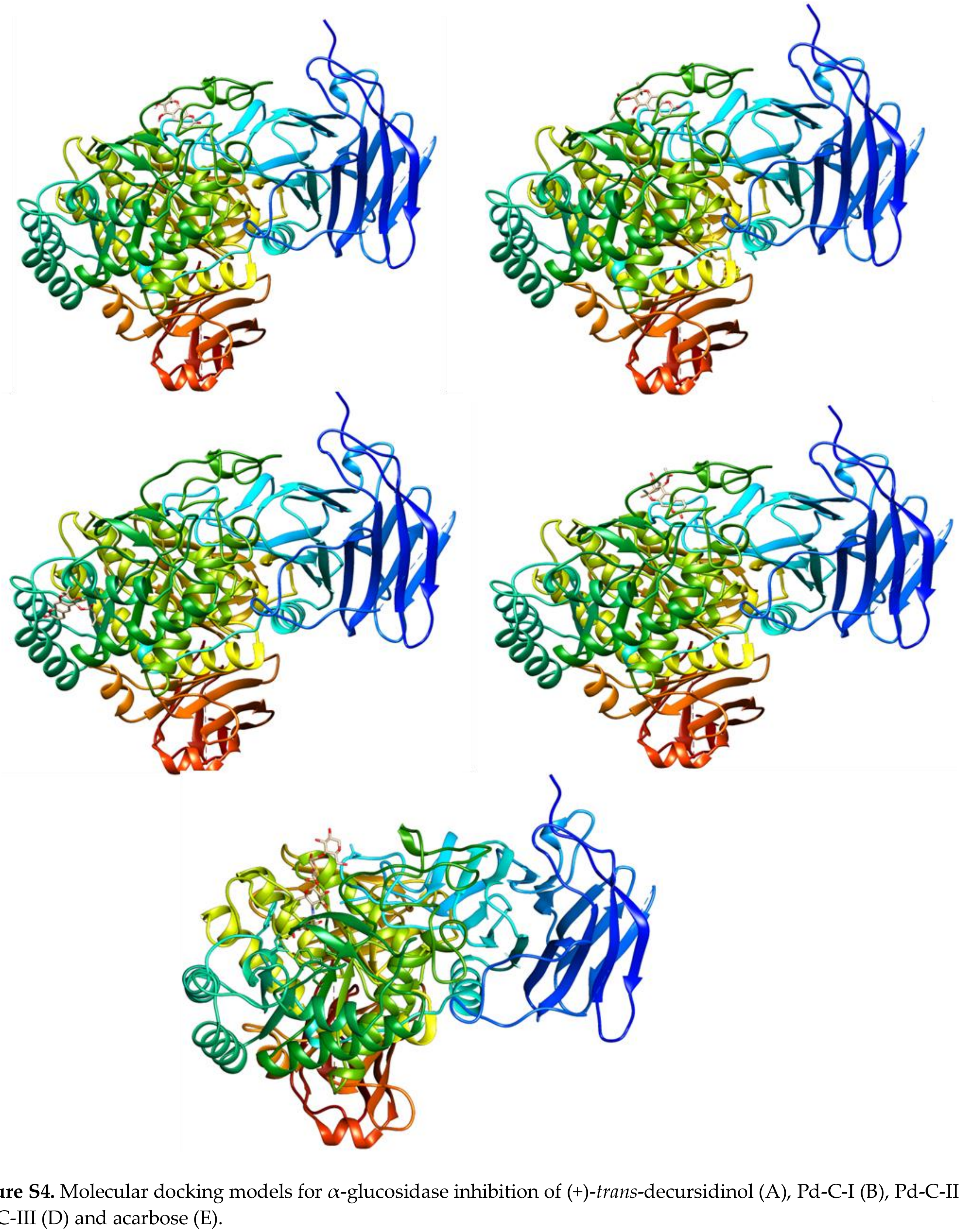

Figure S4. Molecular docking models for α-glucosidase inhibition of (+)-*trans*-decursidinol (A), Pd-C-I (B), Pd-C-II (C), Pd-C-III (D) and acarbose (E).
